# Guide to Interpreting Disease Responses in Chronic Myeloid Leukemia

**DOI:** 10.6004/jadpro.2012.3.4.3

**Published:** 2012-07-01

**Authors:** Ilene Galinsky, Susan Buchanan

**Affiliations:** From Dana-Farber Cancer Institute, Boston, Massachusetts

## Abstract

With the introduction of tyrosine kinase inhibitor (TKI) therapy for chronic myeloid leukemia, the course of the disease has been altered from an acute, rapidly progressive terminal disorder to a serious condition with high remission rates when patients are compliant with long-term treatment. The goal of therapy is to prevent transformation to the accelerated or blast crisis phases, which are associated with poor survival. Knowledge of the appropriate monitoring tests and treatment milestones, as well as the ability to interpret responses, allows advanced practitioners (APs) to effectively communicate key aspects of management to their patients. Monitoring patient responses to TKIs and identifying suboptimal responses early on offer APs the opportunity to reevaluate and adjust therapeutic treatment options. One of the causes of treatment failure is noncompliance; thus, educating patients on the importance of adhering to treatment and identifying reasons for noncompliance are of major importance. Because intolerance to TKIs may lead to discontinuation, frequent monitoring of side effects and response to treatment, open communication, patient education, and careful management are all essential in helping patients remain compliant with therapy. This review discusses the AP’s role in helping patients achieve their best response to TKI therapy and optimize their long-term outcomes.

Chronic myeloid leukemia (CML) is characterized by the increased and unregulated proliferation of mature granulocytes (neutrophils, eosinophils, and basophils) and their precursors. Chronic myeloid leukemia is caused by the development of the Philadelphia chromosome (Ph), formed by the reciprocal translocation between chromosomes 9 and 22 that fuses the ABL gene to the breakpoint cluster region (BCR) gene; the fusion results in the dysregulated expression of the oncogenic BCR-ABL tyrosine kinase protein (Faderl et al., 1999). Chronic myeloid leukemia accounts for 20% of all diagnosed adult leukemias, with approximately 5,000 new cases diagnosed each year in the United States (American Cancer Society, 2010). The incidence of CML increases with age, and it is more likely to occur in men than in women (Rohrbacher & Hasford, 2009). Although the incidence of CML has remained stable over the past 10 years, the prevalence of patients living with the disease has significantly increased due to a rise in overall survival rates provided by tyrosine kinase inhibitor (TKI) therapy.

Untreated, CML can progress through three phases—chronic phase (CP), accelerated phase (AP), and blast crisis (BC)—typically over a period of 3 to 5 years (Vardiman, Harris, & Brunning, 2002). Most CML patients are diagnosed in CP (Vardiman et al., 2002). Potential symptoms are wide-ranging and include lethargy, anemia, splenomegaly, night sweats, and easy bruising. In developed countries, approximately half of patients are asymptomatic; CML is generally diagnosed as a result of routine blood tests (Perrotti, Jamieson, Goldman, & Skorski, 2010). Accelerated phase, deemed an intermediate phase, is more advanced and signals disease progression and the imminence of BC, a critical phase with a median survival of 3 to 6 months (Cortes et al., 2006). Advances in CML treatment and disease-monitoring techniques have changed the course of CML from an often rapidly fatal disease to a serious condition that can be managed in many patients over the long term without transplantation—the only known potential cure (Giles, O’Dwyer, & Swords, 2009; Soverini, Martinelli, Iacobucci, & Baccarani, 2008). This review article focuses on the management of patients with stable, chronic-phase CML (CML-CP).

The most notable advance in the management of CML is the development of the oral TKIs imatinib (Gleevec), nilotinib (Tasigna), and dasatinib (Sprycel). Imatinib, the first TKI introduced for the treatment of newly diagnosed CML patients, inactivates the BCR-ABL kinase to uncouple the many cell processes creating the leukemic state. Tyrosine kinase inhibitor therapies have extended survival and are more tolerable than the previous first-line standard therapy, which consisted of interferon-alfa and cytarabine (O’Brien et al., 2003). Nilotinib and dasatinib were subsequently developed to treat patients resistant to or intolerant of imatinib and are now approved by the US Food and Drug Administration for front-line treatment, based on demonstration of superior efficacy compared with imatinib (O’Brien et al., 2003).

The three TKIs provide options for practitioners to effectively and safely treat their patients with CML. Certain safety issues that have been reported in clinical trials may be of concern to patients with special health issues (Bristol-Myers Squibb, 2010; Novartis Corporation, 2010a, 2010b), and appropriate selection of TKI therapy may be made to fit a patient’s needs. Furthermore, the availability of alternative treatment options is particularly advantageous to the individual who responds poorly or experiences intolerable treatment-related side effects.

Patients with CML are typically managed by a multidisciplinary team that includes the physician, oncology nurses, nurse practitioners, and physician assistants. Successful long-term management of patients with CML requires patient compliance with prescribed treatment and regularly scheduled disease monitoring according to recommended guidelines (Alvarado et al., 2009; National Comprehensive Cancer Network [NCCN], 2011). Health-care providers need to educate patients on the importance of compliance, the potential for common side effects, early reporting of any treatment- or disease-related toxicities, proper administration of medication and critical information relating to the use of concomitant medications, and the importance of keeping scheduled visits to enable assessment of treatment response and compliance over time.

In this article, we describe the commonly used monitoring tests, discuss the responses observed in CML, and review the importance of accurate interpretation of patients’ progress and tolerance to treatment.

## Monitoring Techniques

With appropriate monitoring, patients who are not responding optimally to treatment can be identified promptly and managed in order to minimize the risk for disease progression (Clark, 2009). Allogeneic stem cell transplantation (SCT) has been viewed as the only potential curative treatment for CML, but the advanced age of many patients, potential risk factors, and the availability of a fully matched donor are limitations inherent to this procedure. In fact, the promising survival data from the International Randomized Study of Interferon and STI571 (IRIS) trial—a phase III trial of imatinib (formerly known as STI571) vs. interferon alfa/cytarabine treatment in CML patients—indicated that patients do not require SCT unless they are at high risk for disease progression, e.g., if they have a T315I mutation (Deininger et al., 2009). Therefore, since transformation to AP or BC is associated with poor survival, the goal of therapy is to prevent progression of disease (Robin et al., 2005). Because an early change in therapy for those patients not achieving optimal responses may delay or prevent disease progression, clinicians should be sure to monitor patient response consistently throughout the course of treatment.

Recommendations for monitoring patient response to treatment are found in guidelines developed by the NCCN and the European LeukemiaNet (ELN; Baccarani et al., 2009; NCCN, 2011). The primary goal of the NCCN, which represents an alliance of 21 of the world’s leading cancer centers, is to improve quality and effectiveness of care provided to patients with cancer through the use of NCCN Clinical Practice Guidelines in Oncology (NCCN Guidelines®). The NCCN Guidelines® are developed through an explicit review of the evidence integrated with expert medical judgment and recommendations by multidisciplinary panels from NCCN member institutions. The ELN is the European counterpart to the NCCN. Its guidelines are used more commonly by practitioners in European countries.

The NCCN Guidelines and the ELN guidelines recommend scheduled monitoring of patients with CML, using three sequential parameters: hematologic, cytogenetic, and molecular testing; see Table 1 (Baccarani et al., 2009; NCCN, 2011).

**Table 1:**
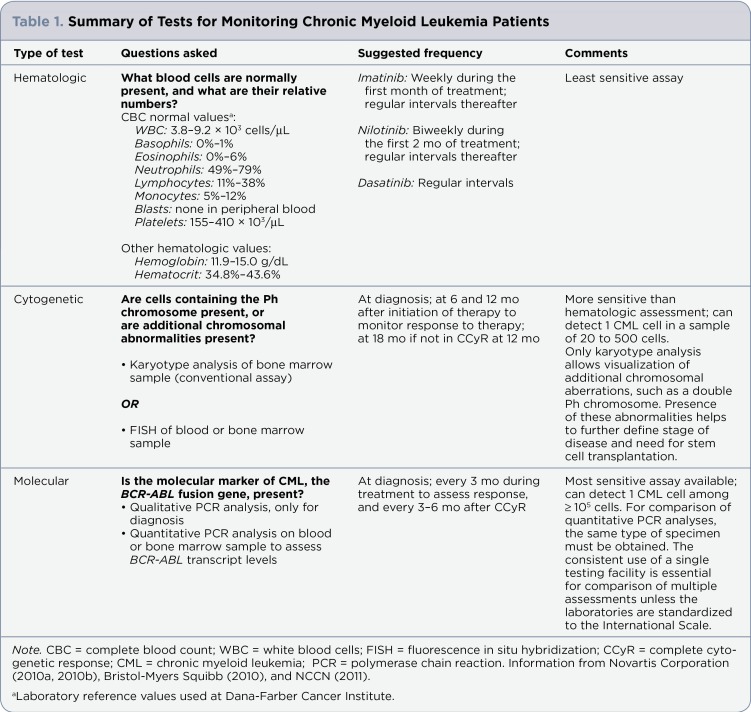
Summary of Tests for Monitoring Chronic Myeloid Leukemia Patients

## Defining Treatment Response and Recommended Schedule for Monitoring

### Hematologic Monitoring

Hematologic analysis is accomplished by obtaining a complete blood count (CBC) with a manual differential. The initial CBC of the CML patient is characterized by abnormally high levels of white blood cells, including leukemic blast cells, promyelocytes, myelocytes, basophils, and neutrophils. Healthy individuals do not have blast cells, promyelocytes, or myelocytes in their peripheral blood, so an early goal of treatment is the eradication of these cell types. The normalization of blood counts is the first response to treatment; hematologic responses are typically observed within 4 weeks of initiating TKI therapy (see Table 2). Frequent monitoring for hematologic response is recommended during early treatment: weekly or biweekly during the first month of treatment (Novartis Corporation, 2010a; Bristol-Myers Squibb, 2010) or biweekly until response is observed (Baccarani et al., 2009), and at regular intervals thereafter, such as every 3 months or as directed in your practice. The NCCN Guidelines are less specific on when or how often to assess hematologic response, but a complete hematologic response is expected by 3 months (NCCN, 2011).

**Table 2:**
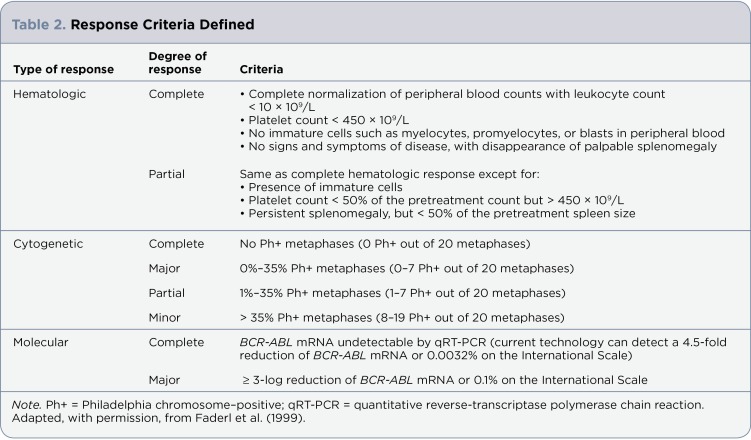
Response Criteria Defined

### Bone Marrow Cytogenetics

Cytogenetic analysis, which is performed with conventional karyotype analysis using bone marrow aspirate, reports the percentage of cells containing the Ph chromosome. A minimum of 20 dividing cells (metaphases) is required for an adequate sample. Karyotype analysis is recommended at baseline to confirm the diagnosis and to rule out other chromosomal abnormalities, such as a double Ph chromosome or an additional aberration that may predict poor outcome (Sadamori, Matsunaga, Yao, Ichimaru, & Sandberg, 1985). Bone marrow cytogenetics is preferred for the initial workup, as it can detect chromosomal abnormalities that are not detectable on peripheral blood fluorescence in situ hybridization (FISH; NCCN, 2011). Following diagnosis, cytogenetic analysis is used to measure treatment response. Cytogenetic responses are defined in Table 2; the lowest response measurement is minor cytogenetic response (mCyR), then partial cytogenetic response (PCyR), and finally complete cytogenetic response (CCyR) as the percentage of Ph+ cells, or leukemic burden, decreases. The ultimate goal is the elimination of the Ph+ cells, or a CCyR.

Cytogenetic analysis of a bone marrow or blood sample to monitor response is recommended 6, 12, and 18 months after initiating TKI therapy (Baccarani et al., 2009; NCCN, 2011). Once the patient achieves CCyR, bone marrow cytogenetic analysis need not be repeated unless patients who have not achieved a major molecular response (MMR) demonstrate increasing BCR-ABL transcript numbers (NCCN, 2011). An alternative method to assess the cytogenetic response is FISH using a peripheral blood sample to detect the presence of the Ph chromosome, particularly when bone marrow collection is not feasible or an inadequate number of metaphases are present (NCCN, 2011). However, it is best to use the same method of assessment at each time point in order to accurately compare responses.

### Molecular Monitoring

In addition to monitoring hematologic and cytogenetic responses, molecular monitoring is an important aspect of measuring response to treatment (see the article on molecular monitoing in CML by Stephanie Bauer and Edie Romvari in the May/June 2012 issue of JADPRO [2012; 3:151–160] ). With the effectiveness of TKIs in eliminating the Ph chromosome, more sensitive tests to quantify minimal residual disease are needed. Because the Ph chromosome produces an abnormal, constitutively activated BCR-ABL tyrosine kinase that is linked to malignant transformation, the presence of BCR-ABL mRNA serves as a surrogate marker of disease activity (Pasternak, Hochhaus, Schultheis, & Hehlmann, 1998). Molecular monitoring, or the analysis of BCR-ABL mRNA, is performed using polymerase chain reaction (PCR) technology, and both qualitative and quantitative assessments are available. It is important to distinguish qualitative (which simply indicates the presence or absence of BCR-ABL) from quantitative (which measures the level of BCR-ABL transcripts).

Advanced practitioners should ensure that the correct PCR method is ordered and reported on test results, as selecting the incorrect PCR analysis method hampers the accurate monitoring of patient response and disease burden. Furthermore, it is critical to consistently provide the same specimen type (bone marrow or blood sample) and use the same testing facility in order to compare sequential measurements to ensure that each sample is assessed with the same processing and internal controls, allowing more accurate comparison between results from different time points.

While qualitative analysis can be used at diagnosis, it is not useful for monitoring response to treatment over time. Quantitative assessments using a modified PCR procedure, real-time quantitative PCR (qRT-PCR), measure BCR-ABL transcript levels in peripheral blood or bone marrow. Using peripheral blood as the specimen source is more convenient and less painful for patients. Quantification of the BCR-ABL mRNA transcript allows practitioners to monitor BCR-ABL transcript levels over time and to detect minimal residual disease (Zhang et al., 2007). As the BCR-ABL transcript levels decrease, the burden of leukemic cells decreases. The development of an international scale that has been validated using molecular response results from many laboratories has led to the standardization of testing methods and reporting across academic and private laboratories (Branford et al., 2006; Branford et al., 2008; Hughes et al., 2006). Molecular response goals—MMR (³ 3-log reduction of BCR-ABL levels) and complete molecular response (CMR, or undetectable BCR-ABL levels beyond a specified sensitivity of the assay)—are described in Table 2. Graphic representation of BCR-ABL values over time can be used as an educational tool to help patients visualize their response and reinforce the importance of adhering to treatment and routine monitoring.

Established guidelines recommend qRT-PCR at diagnosis to establish baseline BCR-ABL transcript levels (NCCN, 2011). Thereafter, qRT-PCR is recommended every 3 months to assess response to therapy, and every 3 to 6 months when a CCyR is achieved (NCCN, 2011). An increase in BCR-ABL levels (typically defined as a 1-log rise) in patients who have achieved an MMR indicates that the test should be repeated. Increasing BCR-ABL levels can alarm patients; advanced practitioners can reassure patients that rising BCR-ABL levels are not an immediate cause for concern or absolute indication of loss of response, as BCR-ABL levels fluctuate over time and can be affected by multiple factors. With confirmation of increased BCR-ABL levels, molecular monitoring should be repeated every 1 to 3 months, and testing for additional mutations (e.g., T315I) should be considered while continuing TKI treatment (NCCN, 2011). Mutation analysis by DNA sequencing is most commonly performed in patients with a suboptimal response, in patients who have lost their response, and in patients who have transformed to AP or BC (Baccarani et al., 2009; NCCN, 2011). Most importantly, changes in TKI therapy are not made based on elevated BCR-ABL levels, but on loss of either hematologic or cytogenetic response.

## Response Milestones

To help clinicians determine the best course of treatment based on responses at a given time point after initiating TKI therapy, the NCCN Guidelines have proposed milestone responses. Table 3 outlines the recommended management for patients according to their responses at specific time points during treatment. However, before changing treatment for patients failing to achieve milestone responses, advanced practitioners should evaluate patient compliance and drug-drug interactions (NCCN, 2011).

**Table 3:**
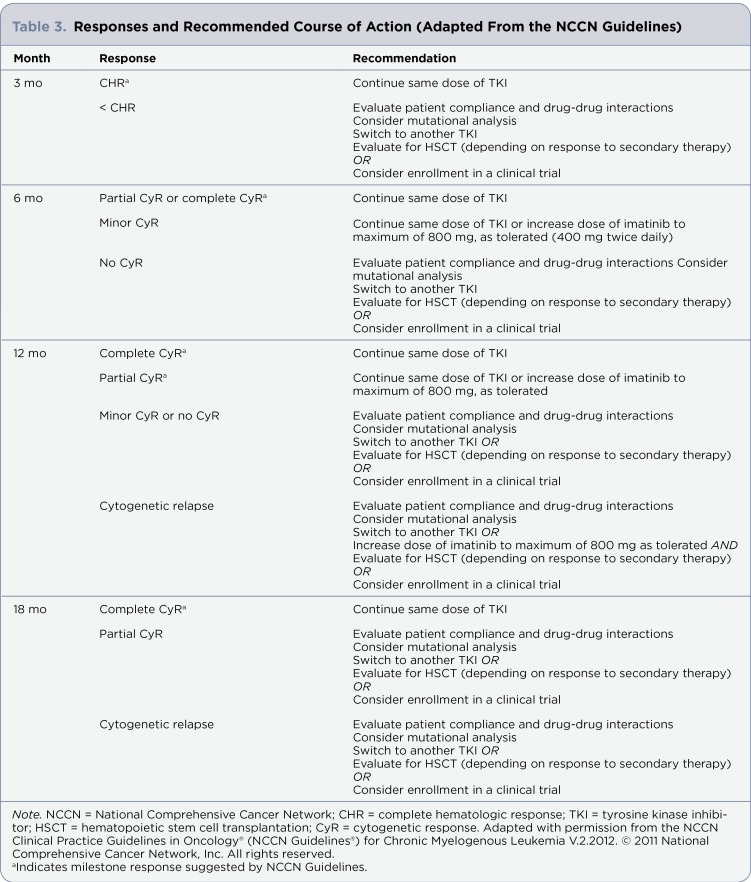
Responses and Recommended Course of Action (Adapted From the NCCN Guidelines)

## Implications of Response

We are continuing to better understand the relevance of achieving milestone responses. In the IRIS study using imatinib for newly diagnosed CML patients, early CCyR (at 6 months) predicted long-term progression-free survival (PFS). At 6 years, the rates of PFS for patients who achieved a CCyR and PCyR at 6 months were 91% and 85%, respectively, vs. 58% and 59% for patients who achieved a minor cytogenetic remission and no cytogenetic remission, respectively (Hochhaus et al., 2009). In addition to increased overall survival (OS), patients who achieved a CCyR were less likely to progress to AP or BC (Hochhaus et al., 2009). Lastly, patients who did not achieve CCyR by 6 months after initiation of therapy had a decreased probability of eventually achieving CCyR and an increased probability of experiencing a poor outcome, defined as progression to AP or BC, loss of complete hematologic response, loss of mCyR, or death (Quintás-Cardama et al., 2009).

The implications of molecular response are also becoming clearer. Analysis of IRIS trial data at 5 and 7 years of follow-up indicated that patients who achieved CCyR and early MMR (12 or 18 months) were less likely to lose CCyR than were patients who had achieved CCyR but no MMR (Hughes et al., 2010). The data also showed that patients who achieved MMR by 18 months were free from transformation to advanced disease (AP or BC) and had a 95% rate of event-free survival (EFS) at 7 years (Hughes et al., 2010). The German study CML-IV has recently demonstrated that imatinib-treated patients who achieved MMR by 12 months had improved rates of OS and PFS at 3 years compared with patients who had not achieved MMR by 12 months: OS, *p* = .0011; PFS, *p* = .0023 (Müller et al., 2010).

These studies suggest that early achievement of MMR is associated with better long-term outcomes. A retrospective analysis of imatinib-treated patients corroborates these results; patients who achieved stable MMR demonstrated increased OS and PFS compared with patients who never achieved MMR (Palandri et al., 2009). Patients treated with front-line nilotinib or dasatinib who achieved MMR did not transform to AP or BC (Kantarjian et al., 2010; Saglio et al., 2010), indicating that MMR may predict better long-term outcomes for patients than the achievement of CCyR without MMR.

These results illustrate an important relationship between a patient’s current response and responses expected at future times and are essential educational points to emphasize with patients through ongoing communication. Therapeutic patient education has been shown to exert a positive effect on health outcomes (Lagger, Pataky, & Golay, 2010). Providing additional context for patients improves their understanding of the necessity for monitoring, compliance to treatment, and interpretation of their own PCR results and may ultimately lead to higher patient satisfaction.

## Undesirable Treatment Responses

Although TKI therapy represents a powerful addition to our armamentarium, not all patients respond to therapy as desired. Undesirable responses include suboptimal response, the development of resistance, intolerance to TKI therapy, and failure (see Table 4). Patients having undesirable responses require careful reconsideration of their treatment regimen and a thorough discussion of treatment options.

**Table 4:**
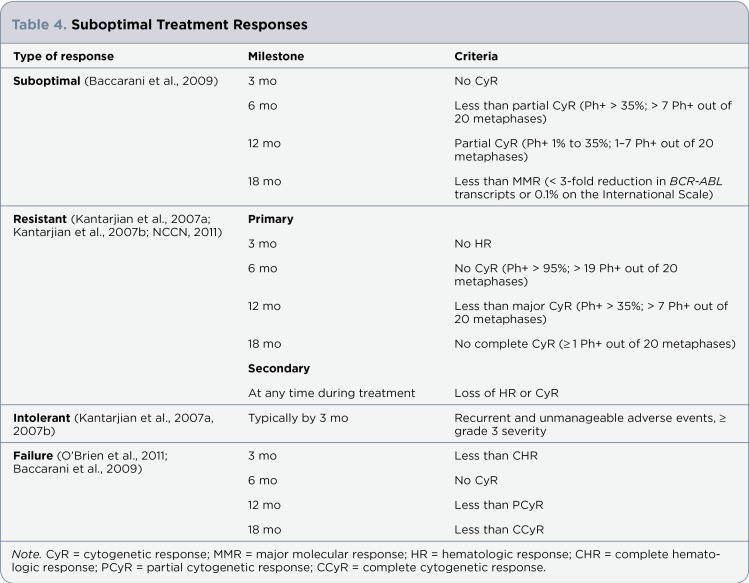
Suboptimal Treatment Responses

### Suboptimal Response

Knowledge of milestone response criteria and an understanding of response implications have helped define suboptimal response. A patient who does not achieve the optimal milestone responses discussed above—that is, complete hemotologic response by 3 months, PCyR by 6 or 12 months, or CCyR by 18 months after initiating TKI therapy—demonstrates a suboptimal response (Baccarani et al., 2009; Marin et al., 2008). In contrast to the NCCN Guidelines, which focus on CCyR as optimal response at 18 months, the ELN guidelines suggest that any response less than MMR at 18 months is suboptimal (Baccarani et al., 2009). In a study of 281 patients treated with imatinib, rates of suboptimal response at 6, 12, and 18 months were compared with long-term outcomes (Alvarado et al., 2009). Patients with a suboptimal response constitute a distinct, albeit heterogeneous, patient population, with a prognosis that depends strongly on the time at which a suboptimal response occurred. The outcome for patients with a suboptimal response at 6 months was similar to that of patients who failed to respond to treatment. In contrast, the outcome for patients with a suboptimal response at 18 months closely resembled that of patients with an optimal response. Therefore, it is important for oncology practitioners to be familiar with the optimal disease milestones to improve their ability to assess their patients’ responses and identify patients who are at increased risk for poor long-term outcomes.

Identifying patients with a suboptimal response is important for clinical decision-making, because it is unclear whether such patients benefit from continuing with their current therapy. Practitioners may consider continuing current TKI therapy at the same dose or increasing the imatinib dose to 800 mg as tolerated (NCCN, 2011). Nonetheless, practitioners may adopt a “wait and see” strategy with a patient with CML-CP to observe whether MMR is subsequently achieved. The ELN guidelines indicate that if MMR is not achieved, switching to a different TKI should be considered (Baccarani et al., 2009); however, the current NCCN Guidelines do not recognize MMR as a milestone (NCCN, 2011).

At least one modifiable factor, patient compliance, has been associated with reduced response. Poor compliance with therapy was the major reason for not achieving MMR in a study of imatinib-treated CML-CP patients (Marin et al., 2010). In addition, noncompliance may result in loss of response (Hughes & Branford, 2009).

### Resistance

An initial lack of response is termed primary resistance, and these patients are unable to achieve optimal outcomes (Mauro, 2006; Alvarado et al., 2009). Secondary resistance refers to acquired resistance that develops during therapy; such patients achieve a complete hematologic, cytogenetic, or molecular response before suffering a relapse or no further response. Most research in this field has focused on the mechanisms of resistance to imatinib, as this was the first TKI approved for CML. To date, resistance to frontline nilotinib or dasatinib has not been described in the literature due to the lack of long-term data.

The etiology of resistance is commonly divided into BCR-ABL–dependent and BCR-ABL–independent types (Agrawal, Garg, Cortes, & Quintás-Cardama, 2010; Volpe, Panuzzo, Ulisciani, & Cilloni, 2009). The best-understood type of resistance is a form of BCR-ABL–dependent resistance, in which point mutations develop within the kinase domain of the BCR-ABL gene. Point mutations result in structural changes in the BCR-ABL protein that interfere with the binding of imatinib to BCR-ABL (Bixby & Talpaz, 2009; Gorre et al., 2001). Many mutations have been found to cause such resistance, but of particular note is the T315I mutation, which is exceptionally difficult to treat; none of the approved TKIs is effective against this mutation (Gorre et al., 2001). However, agents that target T315I are in active clinical trials. Patients can develop multiple mutations within the BCR-ABL gene, which causes more pronounced resistance (Gorre et al., 2001). Amplification and overexpression of the BCR-ABL gene can also contribute to resistance (Agrawal, Garg, Cortes, & Quintás-Cardama, 2010; Volpe et al., 2009).

BCR-ABL–independent resistance to TKIs is expected to be less frequent; this is less well understood, and several mechanisms have been suggested (Agrawal, Garg, Cortes, & Quintás-Cardama, 2010). These include differences in expression levels of the transporter proteins responsible for the import of imatinib into cells, or its efflux from cells, as well as pharmacokinetic considerations, e.g., bioavailability (Hegedus et al., 2002; le Coutre et al., 2004; White et al., 2006; Volpe et al., 2009).

Various options are available for patients who develop resistance (Agrawal, Garg, Cortes, & Quintás-Cardama, 2010). Following imatinib failure, higher imatinib doses may be used, or imatinib may be combined with other agents (e.g., interferon-alfa). However, data on combination therapies are limited and in the early phases of study. Current NCCN Guidelines recommend switching to an alternate TKI (NCCN, 2011). Although all TKIs broadly share the same mechanisms of action, nilotinib and dasatinib have substantially greater in vitro potency than does imatinib and are efficacious against many imatinib-resistant BCR-ABL mutations (Agrawal, Garg, Kantarjian, & Cortes, 2010; Jabbour et al., 2011).

### Intolerance to Treatment

Patient intolerance to TKIs can lead to interruption or discontinuation of treatment (Druker et al., 2006). Specific definitions for intolerance have been applied in each clinical trial, but, in general, intolerance is recognized when a patient develops an adverse event (AE), typically of grade 3 or 4 severity, that cannot be managed with symptomatic relief or through dose reduction. In addition, intolerance may be acknowledged when low-grade AEs interfere with a patient’s quality of life.

In the pivotal IRIS study, approximately 4% of patients discontinued imatinib at 6 years due to AEs (Hochhaus et al., 2009). The most frequently reported AEs with imatinib therapy are mainly hematologic, including neutropenia and thrombocytopenia, and are noted early during treatment. Common nonhematologic AEs include rash, liver toxicity, fluid retention, gastrointestinal intolerance, and musculoskeletal complications (O’Brien et al., 2003).

Treatment intolerance can be addressed by switching TKI therapy. For example, imatinib-intolerant patients may be switched to nilotinib or dasatinib. Although these agents share some AEs with imatinib, cross-intolerance is infrequent (Kantarjian et al., 2010; Saglio et al., 2010). Selection of therapy can be individualized to the patient. For example, patients prone to fluid retention are better served with nilotinib since pleural effusion is reported relatively frequently in patients treated with dasatinib (Kantarjian et al., 2007a; 2010). For patients unable to comply with food restriction during medication dosing, dasatinib could be a better choice than nilotinib.

## Tolerability and Safety Monitoring

In addition to monitoring response to TKI therapy, patients need to be monitored for TKI-related toxicity, including hematologic and laboratory abnormalities. Patients should be routinely questioned regarding symptoms indicative of TKI-related intolerance or toxicity.

The prescribing information for each TKI contains information regarding suggested monitoring before and throughout the course of treatment. Routine blood tests, including CBC, bilirubin, and liver function transaminases, are recommended for all TKIs. Patients taking imatinib should be weighed and monitored regularly for signs and symptoms of fluid retention or unexpected weight gain (Novartis Corporation, 2010a). Complete blood counts should be monitored weekly for the first month, biweekly for the second month, and periodically thereafter (Novartis Corporation, 2010a).

For nilotinib, CBCs are recommended every 2 weeks for the first 2 months and monthly thereafter (Novartis Corporation, 2010b). Electrocardiograms to assess the QTc at baseline, 7 days after initiation of nilotinib, with any dose changes, and periodically thereafter should be obtained (Novartis Corporation, 2010b). Blood chemistry panels should include assessment of serum lipase and amylase (Novartis Corporation, 2010b).

With regard to dasatinib, CBCs should be performed weekly for the first 2 months and then monthly thereafter, or as clinically indicated, to monitor for cytopenias (Bristol-Myers Squibb, 2010). In clinical trials, fluid retention, including pleural and pericardial effusions, has been reported with treatment in both front-line and second-line settings (Bristol-Myers Squibb, 2010). Patients should be closely monitored for any signs or symptoms that may suggest fluid retention, such as cough or dyspnea, and should be promptly evaluated by exam and chest x-ray if there is clinical suspicion.

Treatment-related toxicity may affect compliance to therapy, which may ultimately lead to decreased response and the increased rate of resistance (Marin et al., 2010; Williams et al., 2010). Ensuring patient compliance is a critical component of the long-term management of CML patients. Establishing good communication with patients early in treatment allows practitioners to closely monitor response to therapy, tolerability, and compliance to therapy, and proactive questioning of patients regarding treatment-related toxicity is an important aspect of patient management. As discussed previously, there are several strategies to manage treatment-related toxicity, and for the patient unable to continue treatment due to intolerance, switching to an alternate therapy may be indicated (NCCN, 2011). Active management of AEs may improve patient compliance, leading to improved responses (Giles et al., 2009).

## Role of the Advanced Practitioner

The advanced practitioner is an integral member of the multidisciplinary team caring for the CML patient and plays a critical role in educating patients about CML throughout the course of their disease. Advanced practitioners are the key resource providing patients information concerning their diagnosis, treatment, and monitoring. Because response to treatment can be a source of confusion and concern to patients, advanced practitioners help patients understand their treatment responses and test results. Discussing monitoring results and treatment response over time can improve patients’ understanding and involvement in the management of their disease. Additionally, advanced practitioners are in the best position to assess and encourage patient treatment adherence and to recognize and manage AEs.

## Conclusion

The essential components of CML management include support and education during diagnosis, monitoring, and treatment. Familiarity with the type and recommended scheduling of monitoring tests and the ability to interpret responses allow oncology practitioners to effectively communicate key aspects of management to their patients. The principal assessments used in monitoring responses in patients with CML include hematologic, cytogenetic, and molecular testing, in order of increasing sensitivity. Molecular monitoring is also more selective, as it measures the causative agent of disease, the BCR-ABL transcript. Patients with CML may respond suboptimally or may become treatment-resistant or treatment-intolerant. In such instances, early identification of suboptimal response allows practitioners to reassess the treatment strategy.

Advanced practitioners play a vital role in ensuring that patients appreciate the importance of regular monitoring and the correlation between treatment response and long-term outcomes. Educating patients about their disease and the importance of monitoring milestones, as well as effectively communicating, are both critical in gaining patient trust and confidence. These essential factors rely heavily on establishing a good relationship between the health-care provider and the patient and ultimately affect patient outcomes.

## Acknowledgments

Financial support for medical editorial assistance was provided by Novartis. We thank Nathalie Acher, MD, and Patricia Segarini, PhD, of Percolation Communications LLC for their medical editorial assistance.

### Disclosure

Ilene Galinsky has served on advisory boards for Novartis, Bristol-Myers Squibb, and Celgene. Susan Buchanan has served on advisory boards for Novartis, Bristol-Myers Squibb, and Celgene.
